# Supplementary material for: Differential Effects of Post-translational Modifications on the Membrane Interaction of Huntingtin Protein
Source: ACS Chem Neurosci. 2024 May 16;15(12):2408–19. doi: 10.1021/acschemneuro.4c00091 (PMC11191595; doi:10.1021/acschemneuro.4c00091)
Supplement: Supplementary file 1 — cn4c00091_si_001.pdf [file cn4c00091_si_001.pdf]

Supporting Information

Differential Effects of Posttranslational Modifications on the Membrane Interaction of Huntingtin Protein

Zhidian Zhang[1,2]*, Charlotte Gehin[1*], Luciano A Abriata[2], Matteo Dal Peraro[2**], Hilal Lashuel[1**]

1. Laboratory of Molecular and Chemical Biology of Neurodegeneration, School of Life Sciences, Institute of Bioengineering, Ecole Polytechnique Fédérale de Lausanne (EPFL), 1015 Lausanne, Switzerland
2. Laboratory for Biomolecular Modeling, School of Life Sciences, Institute of Bioengineering, Ecole Polytechnique Fédérale de Lausanne (EPFL), 1015 Lausanne, Switzerland

*contributed equally to the work **corresponding

hilal.lashuel@epfl.ch

matteo.dalperaro@epfl.ch

Supplementary material

Peptides	Sequence	Expected Mass (Da)	Isoelectric point
Htt2-17	ATLEKLMKAFESLKSF	1843	8.43
Htt2-17 OxM8	ATLEKL**(Mox)**KAFESLKSF	1859	8.43
AcHtt2-17	**(Ac)**ATLEKLMKAFESLKSF	1885	7.32
Ac-Htt2-17-AcK6	**(Ac)**ATLEK**(Ac)**LMKAFESLKSF	1927	4.15
Ac-Htt2-17-AcK9	**(Ac)**ATLEKLMK**(Ac)**AFESLKSF	1927	4.15
Ac-Htt2-17-AcK15	**(Ac)**ATLEKLMKAFESLKSF**(Ac)**	1927	4.15
Htt1-19	MATLEKLMKAFESLKSFQQ	2230	8.14
Htt1-19-pT3	MA**(p)**TLEKLMKAFESLKSFQQ	2310	5,71
Htt1-19-pS13	MATLEKLMKAFE**(p)**SLKSFQQ	2310	5.71
Htt1-19-pS16	MATLEKLMKAFESLK**(p)**SFQQ	2310	5,71
Htt1-19-pT3/S13	MA**(p)**TLEKLMKAFE**(p)**SLKSFQQ	2390	4,53
Htt1-19-pT3/S16	MA**(p)**TLEKLMKAFESLK**(p)**SFQQ	2390	4,53
Htt1-19-pS13/S16	MATLEKLMKAFE**(p)**SLK**(p)**SFQQ	2390	4,53
Htt1-19-pT3/S13/S16	MA**(p)**TLEKLMKAFE**(p)**SLK**(p)**SFQQ	2470	3,81

Table S1. Sequence, composition and biochemical properties of peptides used in liposome binding assay. Ac: acetylation, p: phosphorylation, Ox:oxidation

class	lipid	shape	Net charge at pH7	Cell localization	%mol in cells	Link with Htt
**mix**	**Brain lipid extract (TBLE)**	mix	0	mix	100%	HD is a Neurodegenerative disease
**sphingolipids**	**Sphingosine-1-phosphate (S1P)**	Inverted cone	-1	ER, PM, mitochondria	<1%	(Di Pardo and Maglione, 2018)
	**ceramides**	Cylindrical	0	ER, PM, Golgi, lysosomes, mitochondria	<5%	Precursor of cerebrosides, SM and GM1
	**cerebrosides**	Cylindrical	0	Golgi, PM	<1%	Precursor of GM1
	**Ganglioside GM1**	Inverted cone	negative	PM outer leaflet, lysosomes	6% in neurons	(Chaibva et al., 2018)
**glycerophospholipids**	**cardiolipin**	Cylindrical	-2	Mitochondria	5-20% in mitochondria	(Kegel et al., 2009)
	**Phosphoinositide-4-phosphate (PI4P)**	Inverted cone	-3	Golgi	<1%	(Kegel et al., 2009)
	**Phosphoinositide-4,5-biphosphate (PI(4,5)P_2)**	Inverted cone	-4	PM inner leaflet	<1%	(Kegel et al., 2009)
	**Phosphatidylglycerol (PG)**	cylindrical	-1	mitochondria	<5%	(Chiki et al., 2017; DeGuire et al., 2018)
	**Phosphatidylserine (PS)**	cylindrical	-1	All organelles	<10%	(Kegel et al., 2009; Michalek et al., 2013)
**sterols**	**cholesterol**	Inserted inside the bilayer	0	All organelles	40%	(Gao et al., 2016; Michalek et al., 2013)

Table S2. Biophysical properties and biological significance of tested lipids.

Lipid class	Studied lipid	liposomes	Lipid composition (%mol)
**mix**	**Brain lipid extract (TBLE)**	**TBLE**	Brain total lipid extract (100%)
**sphingolipids**	**Sphingosine-1-phosphate**	**S1P**	TBLE (50%); Brain sphingosine-1-phosphate (50%)
	**ceramides**	**ceramides**	TBLE (50%); Brain ceramides (50%)
	**cerebrosides**	**cerebrosides**	TBLE (50%); Brain cerebrosides (50%)
	**Ganglioside GM1**	**GM1**	TBLE (50%); Brain ganglioside GM1 (50%)
**glycerophospholipids**	**cardiolipin**	**cardiolipin**	TBLE (50%); 18:1/18:1 cardiolipin (50%)
	**Phosphoinositide-4-phosphate (PI4P)**	**PI4P**	TBLE (50%); Brain PI4P (50%)
	**Phosphoinositide-4,5-biphosphate (PI(4,5)P_2)**	**PI(4,5)P_2**	TBLE (50%); Brain PI(4,5)P_2 (50%)
	**Phosphatidylglycerol (PG)**	**POPG**	POPG (100%)
	**Phosphatidylserine (PS)**	**PS**	TBLE (50%); Brain PS (50%)
**sterols**	**cholesterol**	**cholesterol**	TBLE (50%); cholesterol (50%)

Table S3. Lipid composition of simple liposomes.

Organelle surrogate	Liposomes	Lipid composition (%mol)
**Endoplasmic reticulum** (van Meer et al., 2008)	**ER**	DOPC (60%), DOPE (20%), DOPI (10%), DOPS (5%), cholesterol (5%)
**Mitochondria** (van Meer et al., 2008; Valencak and Azzu, 2014)	**IMM**	DOPC (40%), DOPE (40%), cardiolipin (15%), DOPI (5%)
	**OMM**	DOPC (50%), DOPE (30%), DOPI (10%), DOPS (5%), cardiolipin (5%)
**Golgi apparatus** (Bigay et al., 2003)	**Golgi**	POPC (50%), POPE (19%), cholesterol (16%), POPI (10%), POPS (5%)
**Plasma membrane** (Temmerman and Nickel, 2009)	**PM**	Cholesterol (50%), POPC (12.5%), Brain SM (12.5%), POPE (10%), POPI (5%), **PI(4,5)P_2 (5%),** POPS (5%)
**Synaptic vesicles** (Fusco et al., 2014)	**SV**	50% DOPE (50%), DOPS (30%), DOPC (20%)

Table S4. Lipid composition of liposomes mimicking the lipid composition of organelle membranes.

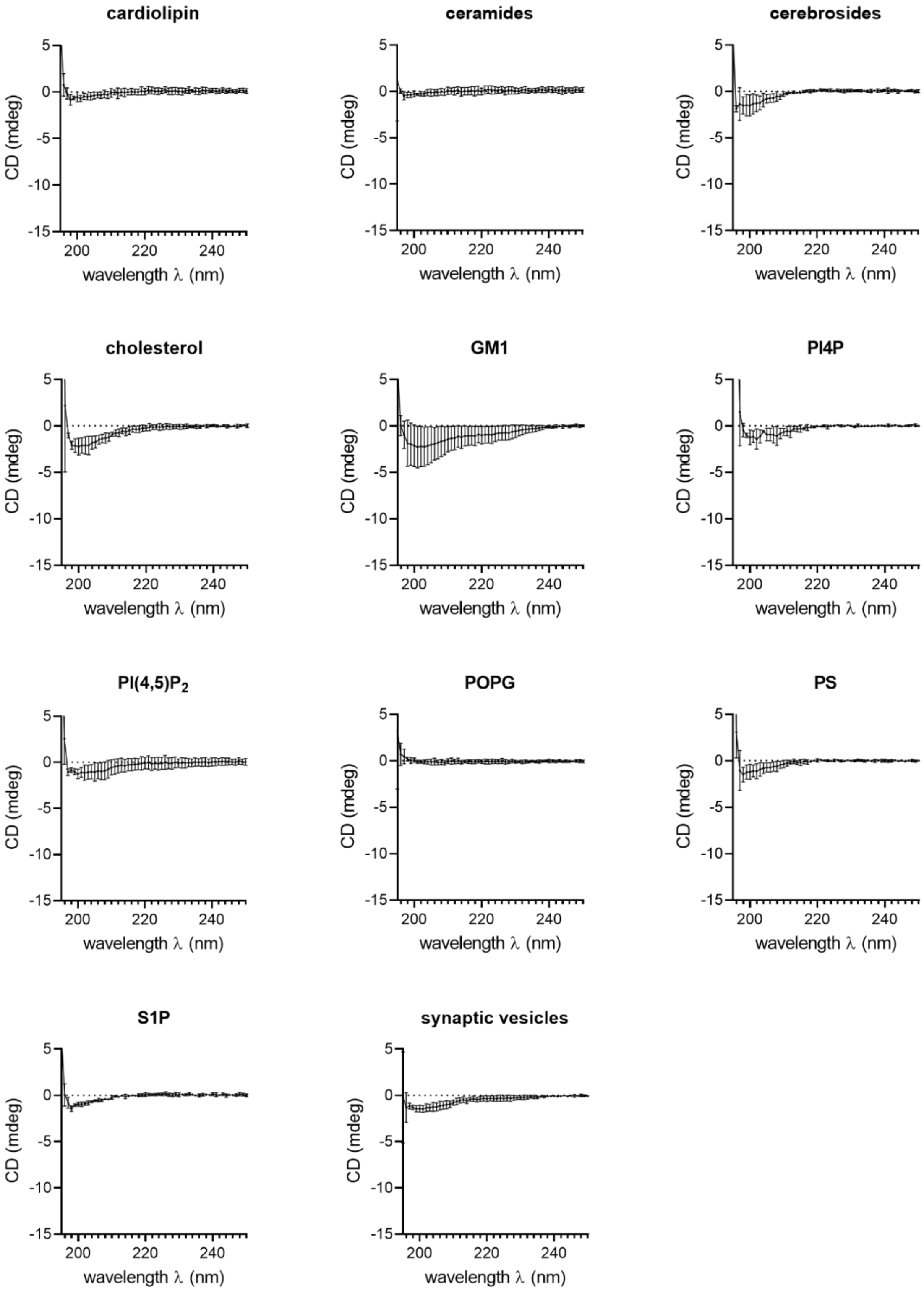
cardiolipin
ceramides
cerebrosides
cholesterol
GM1
PI4P
PI(4,5)P2
POPG
PS
S1P
synaptic vesicles
CD (mdeg)
wavelength λ (nm)
5
0
-5
-10
-15
200
220
240

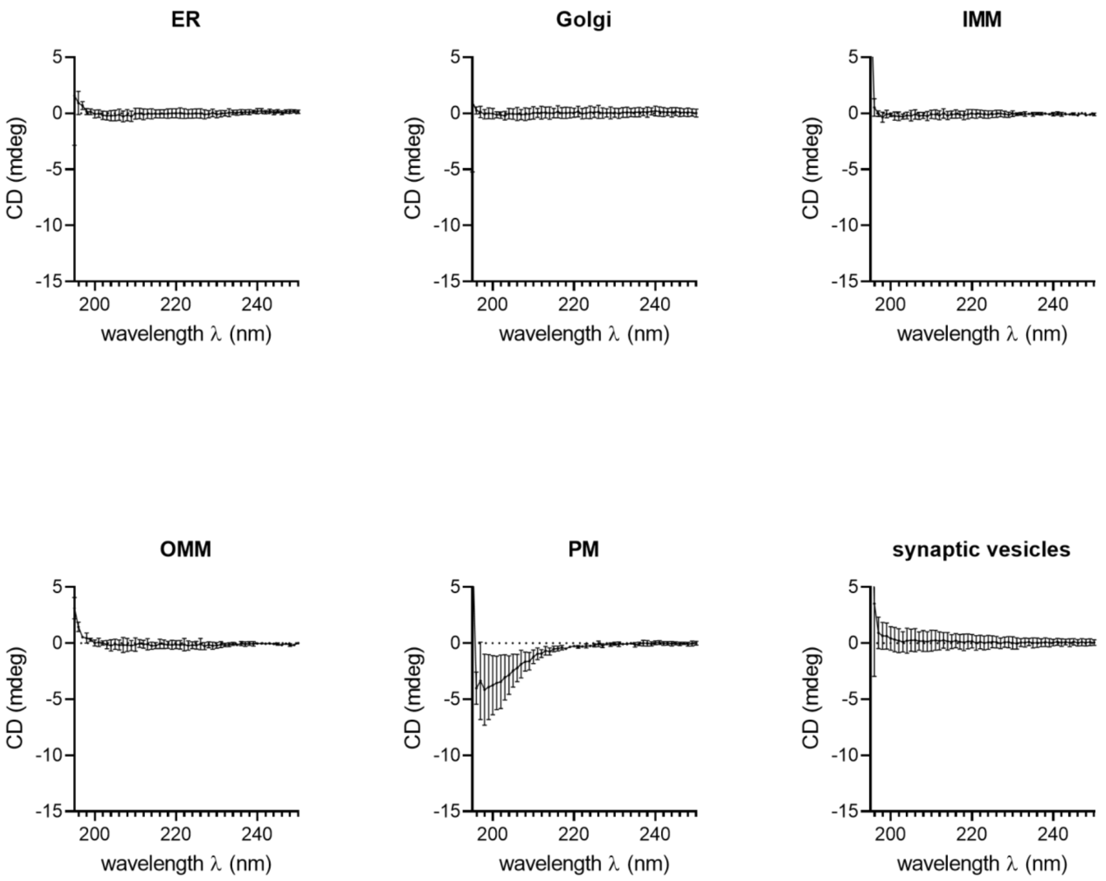

Figure S1. CD spectra of the liposome-containing buffers.

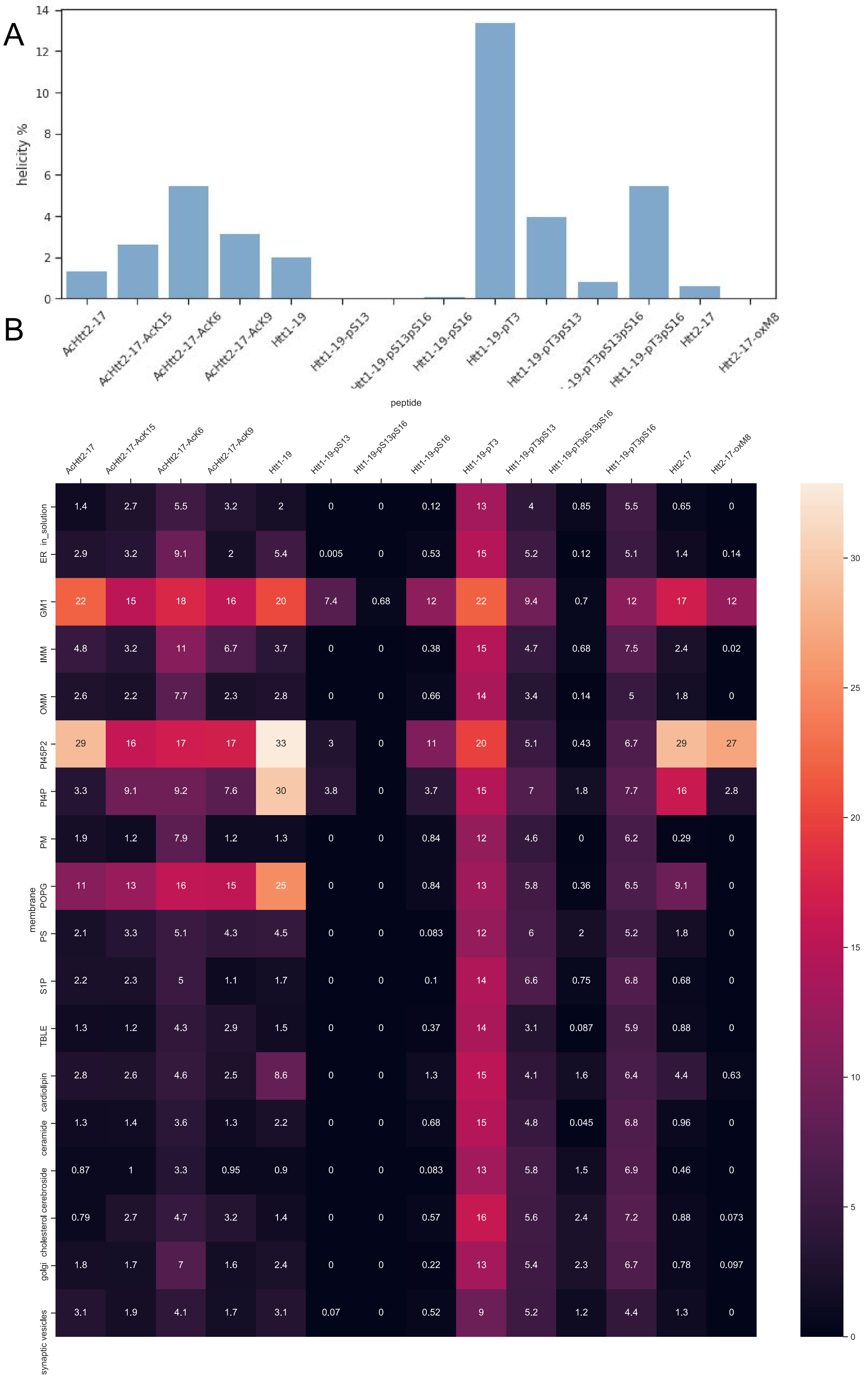
A
14
12
10
8
6
4
2
0
helicity %
AcHtt2-17
AcHtt2-17-AcK15
AcHtt2-17-AcK6
AcHtt2-17-AcK9
Htt1-19
Htt1-19-pS13
Htt1-19-pS13pS16
Htt1-19-pS16
Htt1-19-pT3
Htt1-19-pT3pS13
-19-pT3pS13pS16
Htt1-19-pT3pS16
Htt2-17
Htt2-17-oxM8
peptide
B
AcHtt2-17
AcHtt2-17-AcK15
AcHtt2-17-AcK6
AcHtt2-17-AcK9
Htt1-19
Htt1-19-pS13
Htt1-19-pS13pS16
Htt1-19-pS16
Htt1-19-pT3
Htt1-19-pT3pS13
Htt1-19-pT3pS13pS16
Htt1-19-pT3pS16
Htt2-17
Htt2-17-oxM8
membrane
in_solution 1.4 2.7 5.5 3.2 2 0 0 0.12 13 4 0.85 5.5 0.65 0
ER 2.9 3.2 9.1 2 5.4 0.005 0 0.53 15 5.2 0.12 5.1 1.4 0.14
GM1 22 15 18 16 20 7.4 0.68 12 22 9.4 0.7 12 17 12
IMM 4.8 3.2 11 6.7 3.7 0 0 0.38 15 4.7 0.68 7.5 2.4 0.02
OMM 2.6 2.2 7.7 2.3 2.8 0 0 0.66 14 3.4 0.14 5 1.8 0
PI45P2 29 16 17 17 33 3 0 11 20 5.1 0.43 6.7 29 27
PI4P 3.3 9.1 9.2 7.6 30 3.8 0 3.7 15 7 1.8 7.7 16 2.8
PM 1.9 1.2 7.9 1.2 1.3 0 0 0.84 12 4.6 0 6.2 0.29 0
POPG 11 13 16 15 25 0 0 0.84 13 5.8 0.36 6.5 9.1 0
PS 2.1 3.3 5.1 4.3 4.5 0 0 0.083 12 6 2 5.2 1.8 0
S1P 2.2 2.3 5 1.1 1.7 0 0 0.1 14 6.6 0.75 6.8 0.68 0
TBLE 1.3 1.2 4.3 2.9 1.5 0 0 0.37 14 3.1 0.087 5.9 0.88 0
cardiolipin 2.8 2.6 4.6 2.5 8.6 0 0 1.3 15 4.1 1.6 6.4 4.4 0.63
ceramide 1.3 1.4 3.6 1.3 2.2 0 0 0.68 15 4.8 0.045 6.8 0.96 0
cerebroside 0.87 1 3.3 0.95 0.9 0 0 0.083 13 5.8 1.5 6.9 0.46 0
cholesterol 0.79 2.7 4.7 3.2 1.4 0 0 0.57 16 5.6 2.4 7.2 0.88 0.073
golgi 1.8 1.7 7 1.6 2.4 0 0 0.22 13 5.4 2.3 6.7 0.78 0.097
synaptic vesicles 3.1 1.9 4.1 1.7 3.1 0.07 0 0.52 9 5.2 1.2 4.4 1.3 0
30
25
20
15
10
5
0

C

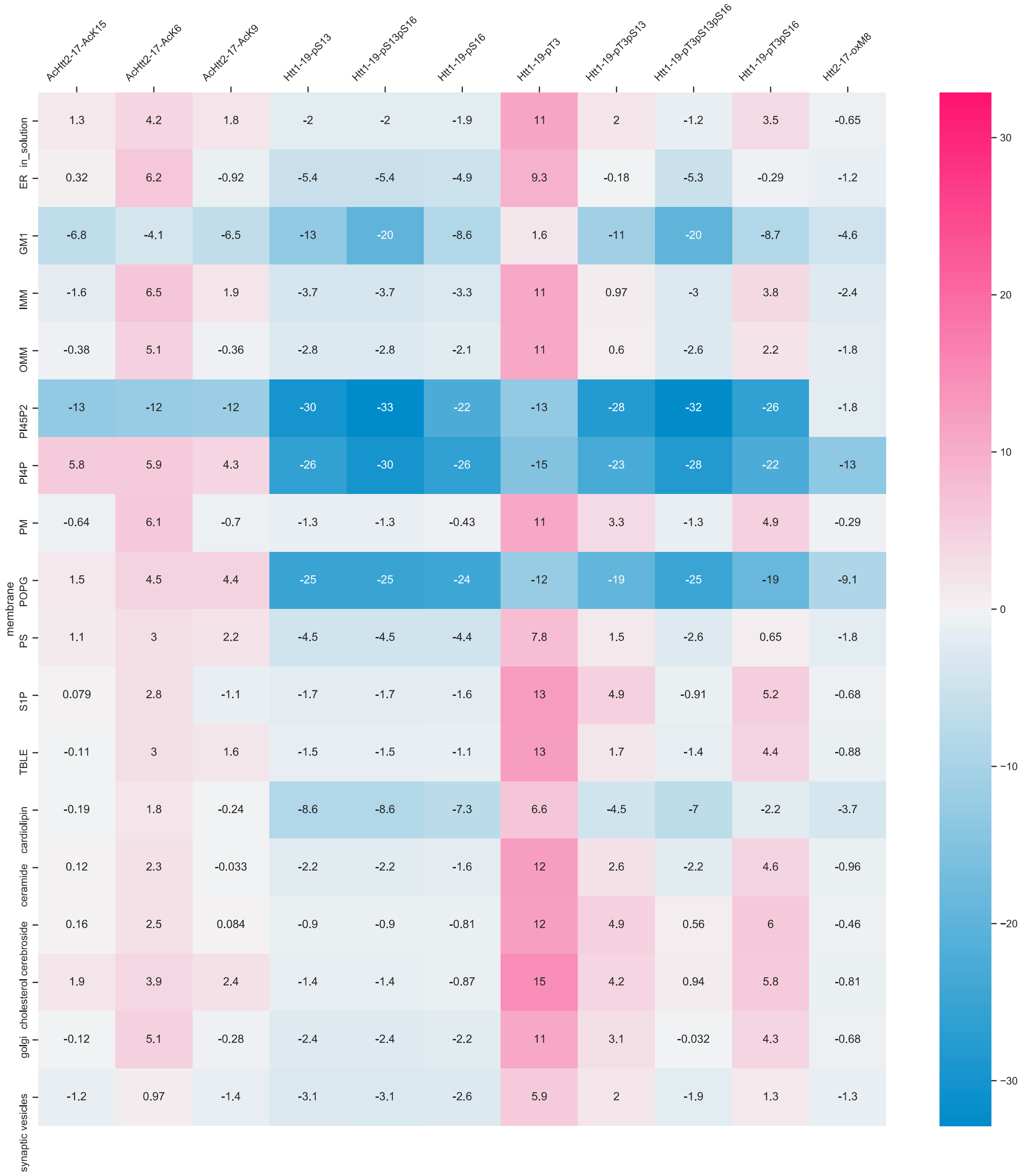

	AcHtt2-17-AcK15	AcHtt2-17-AcK6	AcHtt2-17-AcK9	Htt1-19-pS13	Htt1-19-pS13pS16	Htt1-19-pS16	Htt1-19-pT3	Htt1-19-pT3pS13	Htt1-19-pT3pS13pS16	Htt1-19-pT3pS16	Htt2-17-oxM8
in_solution	1.3	4.2	1.8	-2	-2	-1.9	11	2	-1.2	3.5	-0.65
ER	0.32	6.2	-0.92	-5.4	-5.4	-4.9	9.3	-0.18	-5.3	-0.29	-1.2
GM1	-6.8	-4.1	-6.5	-13	-20	-8.6	1.6	-11	-20	-8.7	-4.6
IMM	-1.6	6.5	1.9	-3.7	-3.7	-3.3	11	0.97	-3	3.8	-2.4
OMM	-0.38	5.1	-0.36	-2.8	-2.8	-2.1	11	0.6	-2.6	2.2	-1.8
PI45P2	-13	-12	-12	-30	-33	-22	-13	-28	-32	-26	-1.8
PI4P	5.8	5.9	4.3	-26	-30	-26	-15	-23	-28	-22	-13
PM	-0.64	6.1	-0.7	-1.3	-1.3	-0.43	11	3.3	-1.3	4.9	-0.29
POPG	1.5	4.5	4.4	-25	-25	-24	-12	-19	-25	-19	-9.1
PS	1.1	3	2.2	-4.5	-4.5	-4.4	7.8	1.5	-2.6	0.65	-1.8
S1P	0.079	2.8	-1.1	-1.7	-1.7	-1.6	13	4.9	-0.91	5.2	-0.68
TBLE	-0.11	3	1.6	-1.5	-1.5	-1.1	13	1.7	-1.4	4.4	-0.88
cardiolipin	-0.19	1.8	-0.24	-8.6	-8.6	-7.3	6.6	-4.5	-7	-2.2	-3.7
ceramide	0.12	2.3	-0.033	-2.2	-2.2	-1.6	12	2.6	-2.2	4.6	-0.96
cerebroside	0.16	2.5	0.084	-0.9	-0.9	-0.81	12	4.9	0.56	6	-0.46
cholesterol	1.9	3.9	2.4	-1.4	-1.4	-0.87	15	4.2	0.94	5.8	-0.81
golgi	-0.12	5.1	-0.28	-2.4	-2.4	-2.2	11	3.1	-0.032	4.3	-0.68
synaptic vesicles	-1.2	0.97	-1.4	-3.1	-3.1	-2.6	5.9	2	-1.9	1.3	-1.3

Figure S2. (A) Helicity of unmodified, acetylated, and phosphorylated Nt17/19 in solution. (B) Averaged helicity of Nt17/19 in the presence of different membranes. (C) Helicity changes resulted from the addition of PTMs.

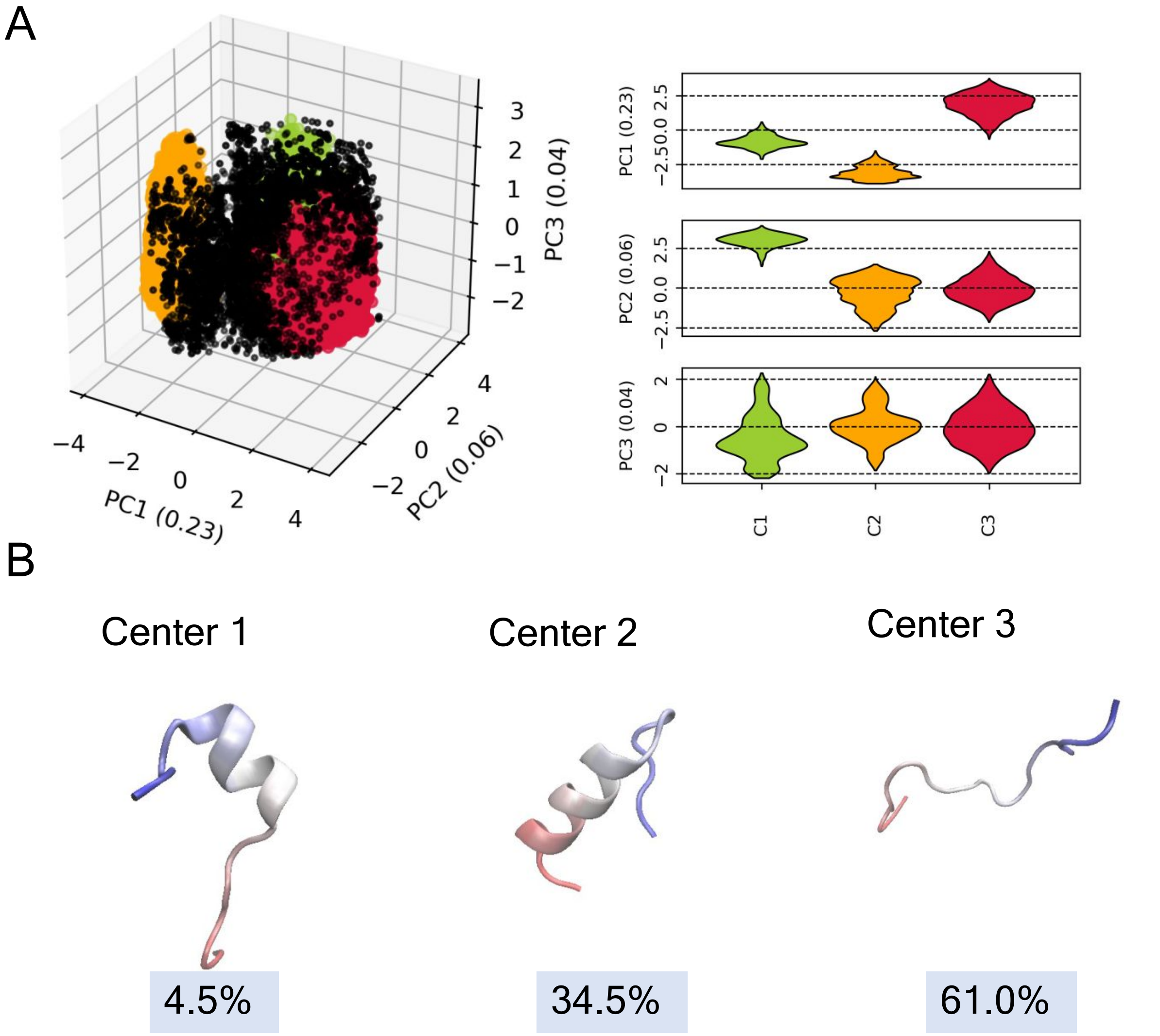

Figure S3. Major conformation extraction from dihedral angle. (A) Clustering result for Nt19 WT based on the first 3 PC. (B) Conformation corresponds to the center of each cluster and the abundance of each cluster. The molecules are colored red to blue from N-term to C-term.

	C1	C2	C3	C4	C5
WT	4.5%	34.5%	61.0%		
	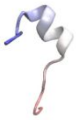	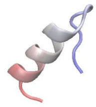	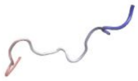		
AcK6	17.6%	21.9%	60.5%		
	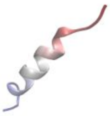	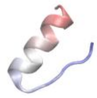	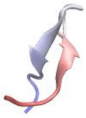	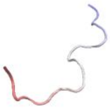	
AcK9	7.3%	6.2%	20.3%	66.2%	
	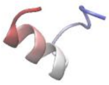	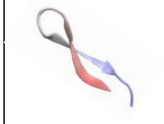	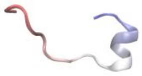	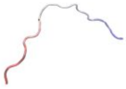	
pT3	3.9%	6.8%	19.4%	46.1%	23.8%
	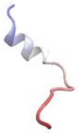	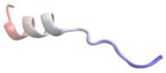	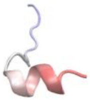	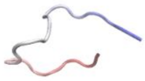	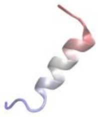

Figure S4. Major conformations and corresponding abundances for WT, AcK6, AcK9, and pT3. The major conformations for each unmodified and modified Nt19 were obtained by clustering based on dPCA of the in-solution simulations. The molecules are colored red to blue from N-term to C-term.

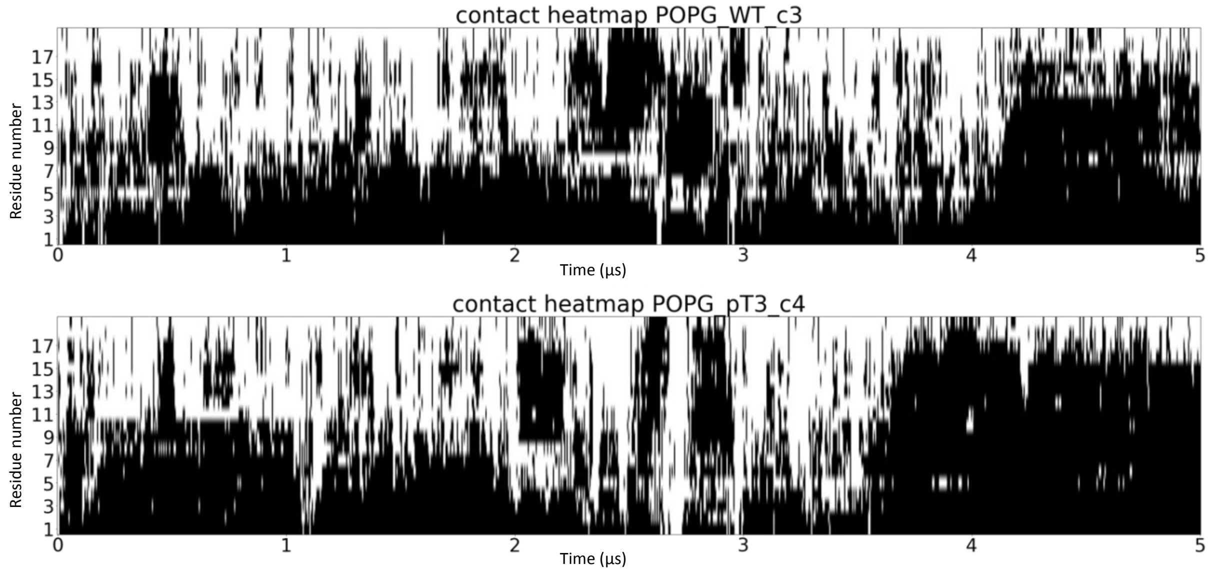

Figure S5. The disordered conformation of pT3 had a similar affinity to the membrane compared to the unmodified Nt19. The contact heatmap for the disordered conformation of pT3 and unmodified Nt19 showed that the disordered pT3 conformation had a similar interaction with the membrane.

Htt2-17

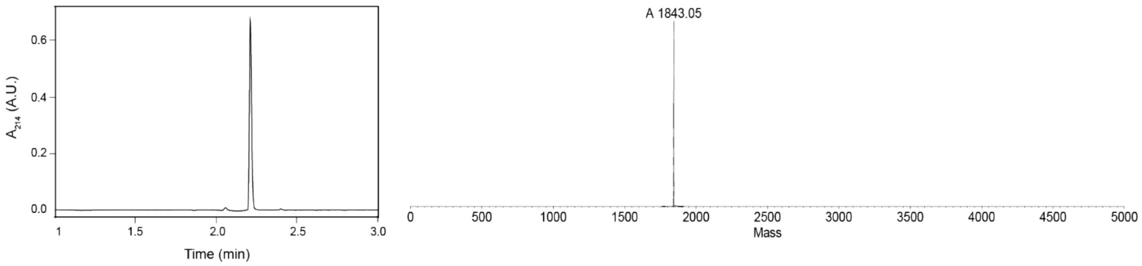

AcHtt2-17

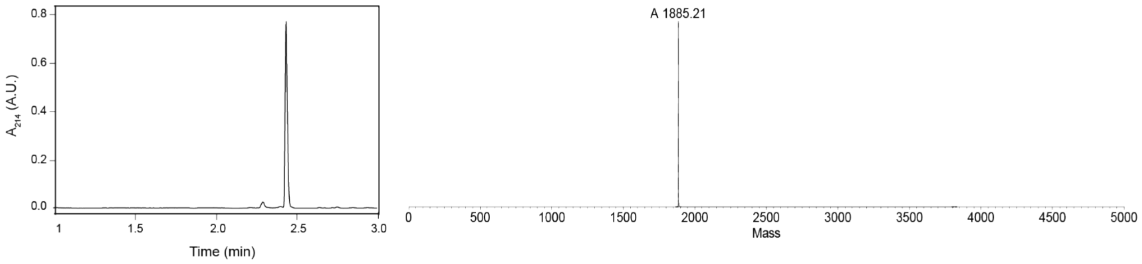

AcHtt2-17-AcK6

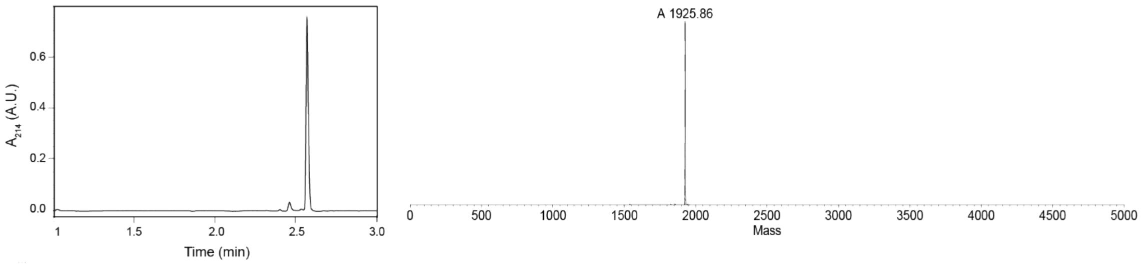

AcHtt2-17-AcK9

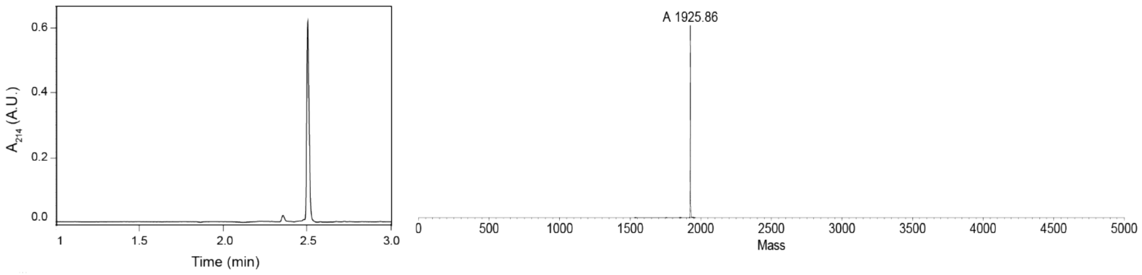

AcHtt2-17-AcK15

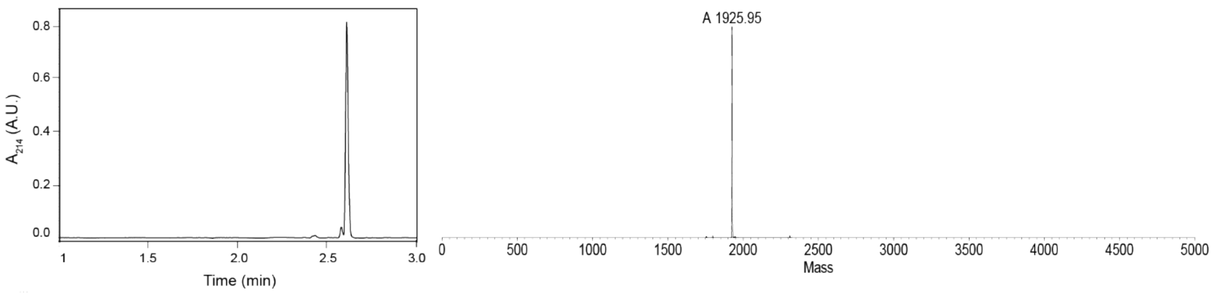

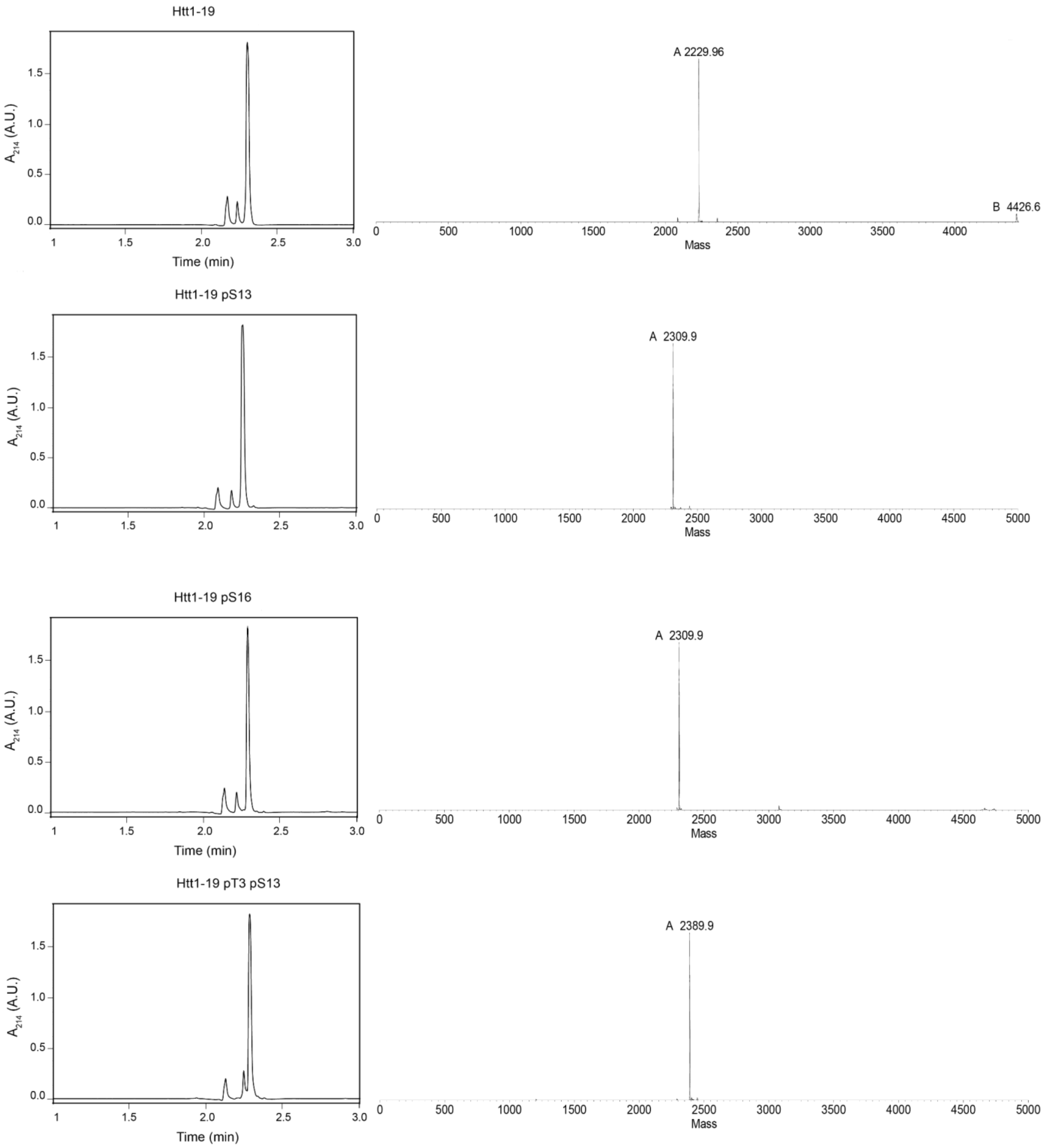
Htt1-19
A 2229.96
B 4426.6
Htt1-19 pS13
A 2309.9
Htt1-19 pS16
A 2309.9
Htt1-19 pT3 pS13
A 2389.9
A214 (A.U.)
Time (min)
Mass

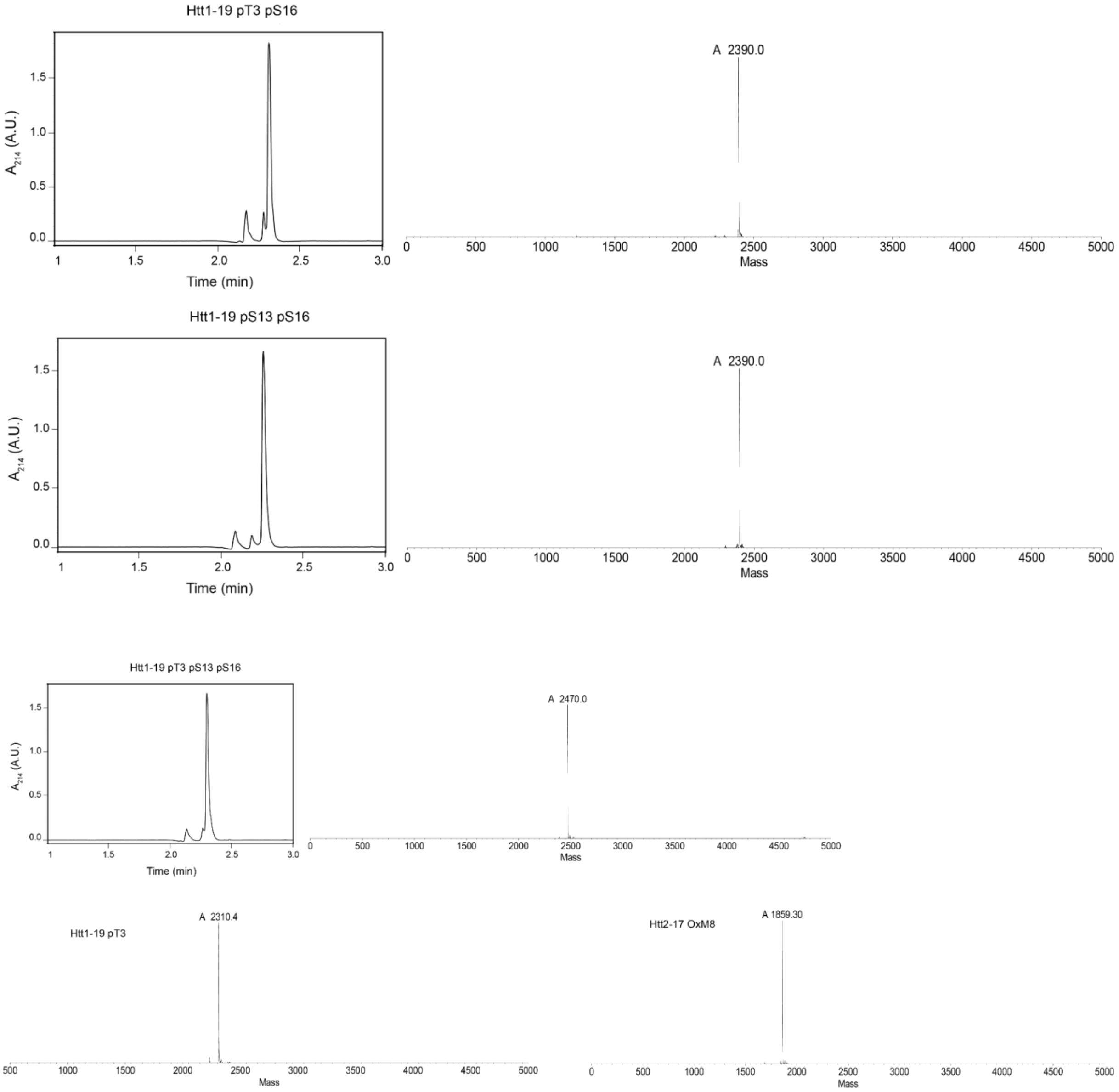

Figure S6. Purity analysis of all peptides from this study assessed by RP-UPLC and LC-MS.

A

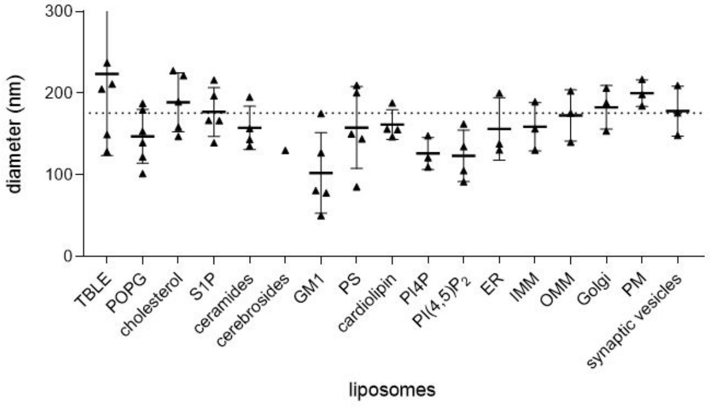

B

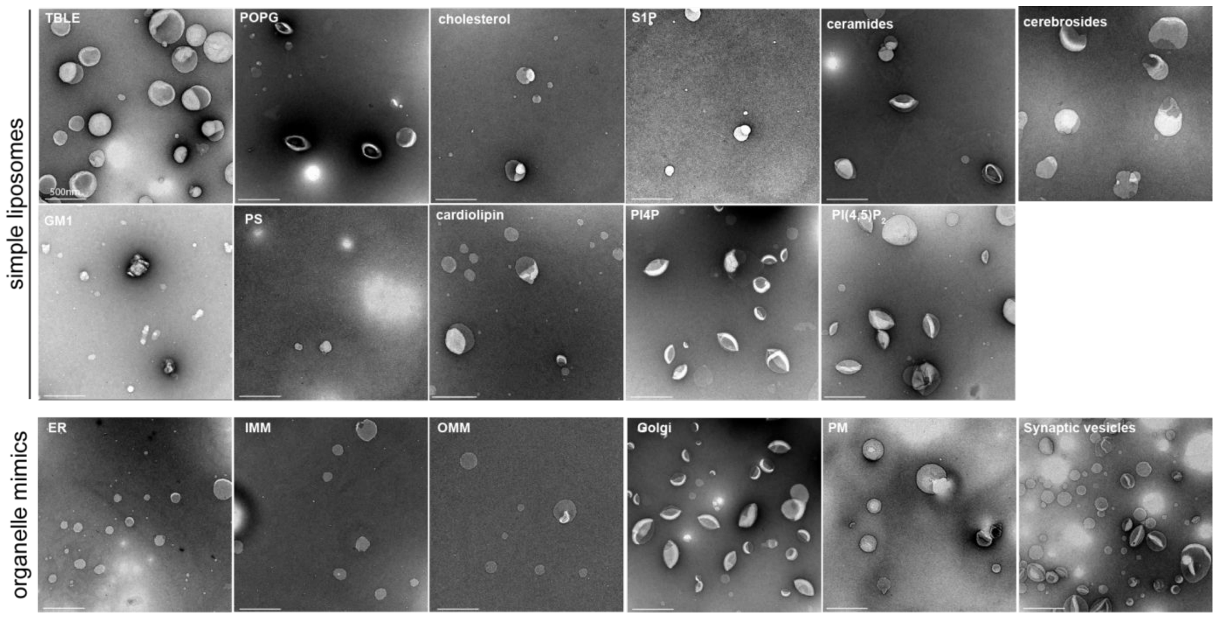

Figure S7. Size distribution of lipid vesicles assessed by A) DLS and B) TEM.
